# Lay descriptions of painful temporomandibular disorders—an international consensus proposal for Global Burden of Disease estimates

**DOI:** 10.1186/s12916-026-04790-3

**Published:** 2026-03-17

**Authors:** A. Lövgren, P. Liv, J. R. Allison, L. Baad-Hansen, E. V. Beecroft, S. Bergman, T. Bijelic, R. Bucci, A. Böthun, N. Christidis, A. Colonna, R. Draghici, M. Drangsholt, J. Durham, M. Ernberg, N. N. Giannakopoulos, D. A. G. Gonçalves, B. Groenke, M. Jusakos, F. Lobbezoo, E. S. P. Lua, G. Lunecke, A. Malysa, D. Manfredini, C. Marpaung, A. Michelotti, E. Moana-Filho, B. Moreno, E. Nilsson, D. R. Nixdorf, L. Nykänen, J. F. Oyarzo, C. C. Peck, C. Penlington, C. Restrepo, R. Rongo, O. Schierz, N. Stanisic, C. M. Visscher, M. Wieckiewicz, X. Xiong, Y.Y. Xu, C. Yanez, B. Häggman-Henrikson, P. Svensson

**Affiliations:** 1 Department of Odontology, Faculty of Medicine, Umeå University, Umeå, Sweden; 2 Department of Public Health and Clinical Medicine, Faculty of Medicine, Umeå University, Umeå, Sweden; 3 School of Dental Sciences, Framlington Place, Newcastle University & Newcastle Hospitals’ NHS Foundation Trust, Newcastle Upon Tyne, NE2 4BW UK; 4 Department of Dentistry and Oral Health, Aarhus University, Aarhus, Denmark; 5 Pacific Northwest, University of Health Sciences School of Dental Medicine, Yakima, WA 98901 USA; 6 Department of Orofacial Pain and Jaw Function, Faculty of Odontology, Malmö University, Malmö, Sweden; 7 Department of Neurosciences, Reproductive Sciences and Oral Sciences, Section of Orthodontics and Temporomandibular Disorders, University of Naples Federico II, Naples, Italy; 8 Division of Oral Rehabilitation, Department of Dental Medicine, Karolinska Institutet, Huddinge, SE-14104 Sweden; 9 School of Dentistry, Department of Medical Biotechnologies, University of Siena, Siena, Italy; 10 School of Dentistry, Department of Prosthodontics, University of Medicine and Pharmacy Carol Davila, Bucharest, Romania; 11 Department of Oral Medicine, University of Washington, Seattle, WA USA; 12 Department of Prosthodontics National &, Kapodistrian University of Athens, Athens, Greece; 13 Department of Dental Materials and Prosthodontics, São Paulo State University (UNESP), School of Dentistry, Araraquara, Brazil; 14 Minnesota Dental Research Center for Biomaterials and Biomechanics, University of Minnesota, Minneapolis, USA; 15 Faculty of Odontology, Orofacial Pain and TMD Program, Universidad Andres Bello, Santiago, Chile; 16 Department of Orofacial Pain and Dysfunction, Academic Centre for Dentistry Amsterdam (ACTA), University of Amsterdam and Vrije Universiteit Amsterdam, Amsterdam, The Netherlands; 17 Faculty of Dentistry, National University of Singapore, Singapore, Republic of Singapore; 18 Department of Experimental Dentistry, Faculty of Dentistry, Wroclaw Medical University, Wroclaw, Poland; 19 Department of Prosthodontics, Faculty of Dentistry, Universitas Trisakti, Jakarta, Indonesia; 20 School of Dentistry, University of Minnesota, Minneapolis, USA; 21 Head and Neck Center, Helsinki University Hospital, Helsinki, Finland; 22 Department of Oral and Maxillofacial Diseases, University of Helsinki, Helsinki, Finland; 23 CES-LPH Research Group, Universidad CES Medellín, Medellín, Colombia; 24 Department of Prosthodontics and Materials Science, University of Rostock, Rostock, Germany; 25 State Key Laboratory of Oral Diseases, National Clinical Research Center for Oral Diseases, West China Hospital of Stomatology, Sichuan University, Chengdu, Sichuan China; 26 The Institute for Health Metrics and Evaluation, University of Washington, Seattle, WA USA; 27 Heidelberg Institute of Global Health, Section for Oral Health, Faculty of Medicine and University Hospital, Heidelberg University, Heidelberg, Germany; 28 Department of Rehabilitation Sciences, KU Leuven, Louvain, Belgium

**Keywords:** Epidemiology, Facial pain, Global Burden of Disease, Health impact assessment, Quality of life

## Abstract

**Background:**

Despite being one of the most common chronic pain conditions, painful temporomandibular disorders (TMDs) are still not included in the Global Burden of Disease (GBD) measures of health loss. A key obstacle is the absence of a lay description that can be used to derive a disability weight reflecting the severity relative to all other diseases’ consequences measured in GBD. This study aims to propose lay descriptions of painful TMDs suitable for use within the GBD framework.

**Methods:**

The process was guided by experts from the Institute for Health Metrics and Evaluation (IHME), USA. A structured consensus process was conducted among international experts, clinicians, and patient representatives in three steps: terminology alignment, roundtable discussions during a workshop, and outcome synthesis. After aligning terminology through introductory lectures, five groups of 8–12 participants reviewed existing lay descriptions for other pain-related disorders. In addition, they discussed lay descriptions and severity states.

**Results:**

No existing descriptions adequately captured the characteristics of painful TMDs. After consensus discussions, the proposed lay description was “Pain in the jaw, face, cheeks, or around the ears, sometimes radiating to the temples or behind the eyes. The pain may be felt as dull, sharp, tense, or stiff, making chewing, talking, or opening the mouth difficult.” Duration, intensity, and frequency were identified as important dimensions linked to severity.

**Conclusions:**

The lay description provides direction on how painful TMDs can be quantified in GBD estimates. Future studies, including those that incorporate patients’ perspectives, are essential to ensure alignment with lived experiences.

**Supplementary Information:**

The online version contains supplementary material available at 10.1186/s12916-026-04790-3.

## Background

### Definition and impact of TMD pain

Painful temporomandibular disorders (TMDs) are the most common cause of chronic orofacial pain (OFP), affecting approximately 1 in 10 adults [1]. They typically manifest as pain in the face, jaw muscles, and/or joints, and surrounding tissues. TMDs are often worsened by jaw function and are associated with impaired oral function, reduced quality of life, and psychological distress [2–4]. According to the internationally accepted classification systems—namely, the Diagnostic Criteria for TMDs (DC/TMD) and the International Classification of Orofacial Pain (ICOP)—the diagnostic criteria for TMD pain are pain experienced within the past 30 days, influenced by jaw function, localized to the temporalis or masseter muscles or the temporomandibular joint, and reported as representative/familiar to the individual during clinical examination [5]. In addition to localized symptoms, painful TMDs have a strong bidirectional association with comorbidities, such as primary headaches, neck pain, and widespread musculoskeletal pain including chronic overlapping pain conditions [6], as well as with negative consequences on health, such as sleep disturbances, anxiety, and depression [7–9]. In this context, painful TMDs are today understood as a condition within a broader spectrum of chronic pain disorders, likely mediated by nociplastic pain mechanisms [10] and with potentially shared vulnerability in pain pathways including genetic susceptibility, environmental, behavioral and emotional risk factors, and sleep disturbances [8]. As one of the most prevalent chronic pain conditions worldwide, painful TMDs impose a substantial burden on both individuals and society [11–14]. Nevertheless, painful TMDs and other OFP conditions are often not represented in major epidemiological datasets and research agendas, contributing to a cycle of invisibility in both clinical and policy contexts.

### Importance of including TMD pain in GBD measures

The Global Burden of Disease (GBD) initiative provides one of the most impactful frameworks for quantifying health loss across populations [15] and has become a cornerstone for global health research and policy through its systematic, standardized estimates of disease prevalence, risk factors, and disability across time and regions. The Institute for Health Metrics and Evaluation (IHME) in Seattle, USA, is the leading organization for producing GBD estimates worldwide. For non-communicable diseases, including those commonly managed in dentistry that are associated with morbidity but not mortality, the disease burden is estimated by a combination of the disease prevalence and a disease-specific disability weight of the years lived with disability [16]. We recently provided the first estimates of disability weights for OFP, indicating that its impact is greater than that of toothache alone and is comparable with that of tension-type headache and other chronic conditions that require daily medication [17]. However, many highly prevalent OFP conditions, including painful TMDs, remain absent from these global assessments [18]. Recognizing and including TMD pain and OFP conditions in GBD measures would yield a more accurate representation of the global burden of pain-related disorders to inform targeted clinical interventions, resource allocation, and health policy planning.

### Prerequisites for including a new condition in the GBD

For a condition to be included in the GBD framework, certain key prerequisites must be met in accordance with IHME guidelines. This includes establishing a standardized case definition, which ensures the condition is consistently identified and communicated, and developing *lay descriptions* to elicit the associated *disability weights* [19]. A lay description is a concise, non-clinical summary of a health state that enables a clear understanding, by the general public, of its functional consequences. Lay descriptions enable the comparison of the impact between diseases. The aggregation of all survey comparisons allows the quantification of the functional consequences of a condition as a “disability weight” [19]. This process is known as valuation and is intended to capture the public perception of health loss (or disease’s burden) for each disease relative to all other diseases in a 0 (no health loss/full health) to 1 (complete loss of health/burden equivalent to death) scale [20]. When valuation is not available, mapping techniques can be used to derive an approximate estimate [16]. Based on general population data, we have mapped a disability weight estimate for TMDs of approximately 0.03 [17], reflecting a level of disability similar to that of tension-type headache (0.037, 95% confidence interval (CI): 0.022–0.057) [21]—a condition that frequently overlaps with TMD—and comparable with that of generic chronic diseases (0.049, 95% CI: 0.031–0.072) [21]. Furthermore, when relevant, the disease severity should be reflected in distinct categories linked to corresponding severity-specific lay descriptions. In this regard, our findings indicated that pain intensity, worrying, limited jaw function, and sleep could be relevant factors to include when grading the severity of TMD pain [17]. However, despite the availability of well-established, evidence-based case definitions for TMD pain via the DC/TMD [5] and ICOP [22], lay descriptions have not been proposed for TMD pain.

Our aim was to develop, discuss, and propose consensus-based lay descriptions of TMD pain suitable for implementation into the GBD framework in collaboration with the International Network for Orofacial Pain and Related Disorders Methodology (INfORM) group of the International Association for Dental, Oral, and Craniofacial Research (IADR) and with patient representatives.

## Methods

The consensus process comprised three key steps: terminology alignment, roundtable discussions in a workshop, and synthesis of the findings. The first step included identifying and aligning terminology with evidence-based and adopted terminology relevant to TMD pain and GBD lay descriptions. This work was guided by experts from the IHME (LO, TV, and YX). The preparations included deciding on the description of the case definition, ensuring alignment with the DC/TMD criteria, and categorizing cases into mild, moderate, and severe. During these discussions, the possibility of using “possible” and “probable” for categorization—which are included in the International Classification of Headache Disorders [23], and used for neuropathic pain conditions [24]—was considered; however, these terms were deemed unsuitable for use in this context, as they are not part of the established case definition criteria for TMD pain. Because of the well-known differences in underlying mechanisms [25], distinctions between “acute” and “chronic” painful TMDs were also considered; however, it was suggested that these terms would not be used, given that they define pain duration rather than severity per se. Moreover, in GBD estimates, the duration of all conditions is estimated by onset and remission data, rather than using more generalized, predefined durations such as the currently accepted cut-off threshold for acute (< 3 months) and chronic pain.

The next step was to share the above proposals and discuss possible content in lay descriptions of painful TMDs. Hence, a satellite symposium was arranged in conjunction with the 105th General Session & Exhibition of IADR 2025, entitled “Joint Action Needed: Integrating Orofacial Pain into Global Burden Measures.” The symposium aimed to identify methodological approaches for estimating the global burden of OFP and to discuss and propose terms to be included in the lay descriptions for TMD pain (Additional file 1). Sponsorship provided by INfORM was combined with co-sponsorship from the Neuroscience Group of IADR. The symposium was open to all IADR members and was announced on the IADR website and to members via INfORM newsletters.

The symposium opened with introductory lectures aimed at providing an understanding of the background for the GBD project, including definitions, terminology, and a common language for TMD pain. The lectures covered the following topics, presented by the authors of this paper: (1) the global burden of pain, including an overview of GBD methodology and the burden of pain conditions (PS); (2) prerequisites for GBD estimates of OFP methodological requirements, including the collection of incidence, prevalence, and risk factor data (MD); (3) disability weights and methods, involving the importance of disability weights and approaches for generating them (ALö); and (4) the patient perspective, discussing the challenges of integrating OFP into healthcare from a stakeholder perspective (SB).

### Development of lay descriptions for painful TMD

A structured multi-step workshop was conducted to develop lay descriptions for TMD pain, building on participant input (Additional file: Table 1). Participants comprised a diverse group of well-established international experts and leading researchers, as well as early- and mid-career researchers and clinicians with extensive experience in diagnosing and managing TMD and pain conditions. The group also included participants from other dental specialties as well as individuals from disciplines outside dentistry (e.g., psychology and physiotherapy). Experts represented multiple continents, cultures, ethnic backgrounds, and country income levels, providing a broad and diverse perspective. Participants were divided into five groups, each led by a chairperson (CV, JD, MD, PS, and ALö). To ensure content expertise and facilitate discussions, each group was co-chaired by a facilitator with prior experience in data extraction following the IHME procedure (NS, EL, BHH, TB, and AB). The workshop concluded with group presentations of the findings.

**Fig. 1:**
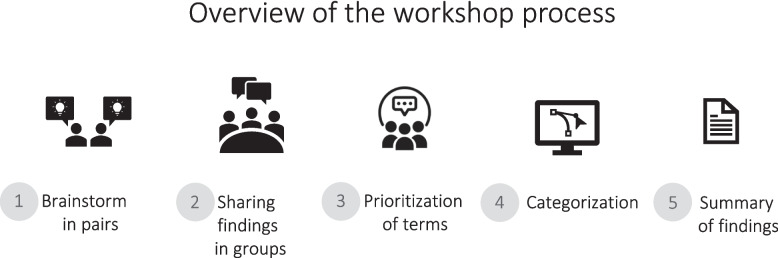
Outline of the workshop process for developing lay descriptions of painful TMDs

In developing the lay description, we aimed to avoid the same terminology as the disease/disorder and to include key signs and symptoms, as well as a grading of the impact on daily living. The lay description was required to be less than 35 words, ideally less than 30, while avoiding medical/dental jargon. Artificial intelligence (AI) tools were not allowed in the first four steps to ensure that the participants’ descriptions reflected only their own perspectives. In the final step, however, participants were allowed to use AI tools to generate and refine lay descriptions. The full process is described below (Fig. 1).

- Step 1: Brainstorming key terms. Participants in the five groups, with 8–12 participants in each group, worked in pairs for approximately 15 min to identify words and phrases describing TMD pain, recording them on separate sticky notes. The suggested terms were then shared and discussed with the group to create an initial descriptor pool. All sticky notes were gathered by the facilitators of the group discussions.
- Step 2: Review of existing lay descriptions**.** Existing lay descriptions for related pain conditions (e.g., neck pain, back pain, toothache) were reviewed to identify adaptable and similar elements for TMD pain. Lay descriptions from related conditions that were also considered suitable for TMD pain were highlighted.
- Step 3: Prioritization and categorization**.** The groups collaboratively selected and prioritized the most relevant terms, organized them into a hierarchical list, and introduced standardized health states as per IHME guidelines: mild, moderate, and severe. Specific descriptors were mapped to severity levels.
- Step 4: Drafting lay descriptions. Using the prioritized terms, preliminary lay descriptions of TMD pain were drafted for each severity level and iteratively refined based on group discussion.
- Step 5: AI-assisted refinement. AI tools were allowed to be used to generate alternative lay descriptions. A detailed description of this step is included later in this piece.

### Synthesis of findings

All the individual works derived from the brainstorming were compiled into a joint list. Similar or overlapping terms from the list were grouped to reduce redundancy. Terms expressing the same concept were merged, although distinctions were maintained where necessary to preserve nuances. Similarly, all the groups’ descriptions of mild, moderate, and severe cases were compiled into joint lists.

Separately from the workshop, we used ChatGPT version 4.0 with our compiled lists and the prompts, “develop lay descriptions as per IHME standards for painful TMD” and “divide into three separate categories including mild, moderate, and severe” to generate suggested lay descriptions for comparison.

## Results

**Fig. 2:**
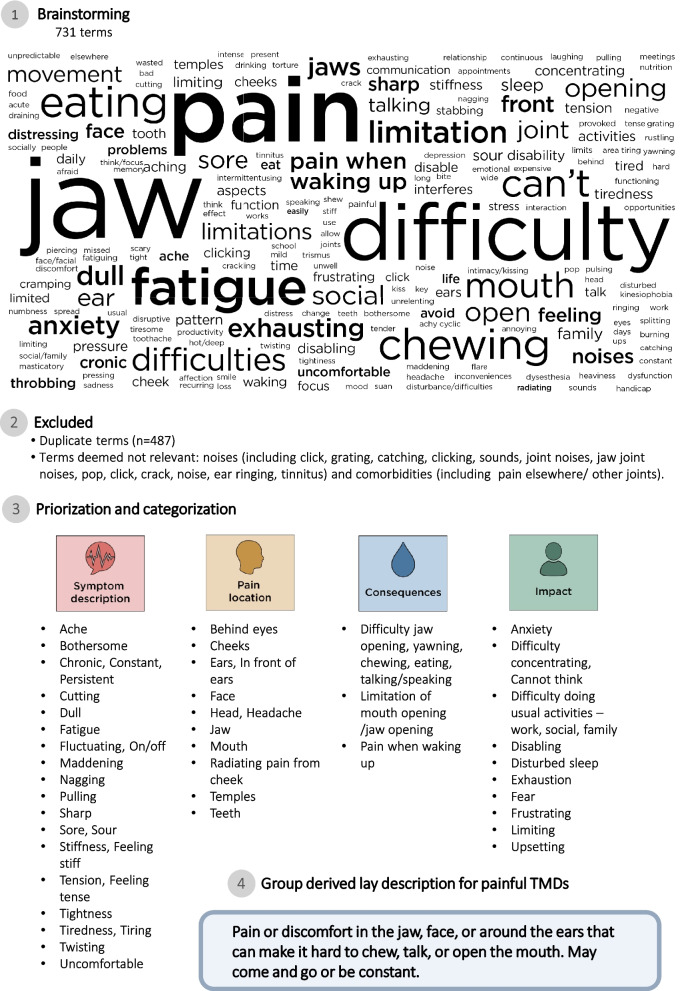
Summary of the workshop process, along with the terms and descriptions derived at each step

The attendees of the symposium included clinicians, researchers, and patient representatives (Table 1). When the existing lay descriptions for other pain conditions were reviewed (Table 2), some were considered partly relevant to TMD pain, such as those for neck pain; however, none were deemed sufficiently inclusive to capture the key components necessary to convey the essence of TMD pain. The five groups derived 718 individual terms or phrases (Supplement 3). After duplicates and terms considered insufficiently relevant to TMD pain were excluded, 231 unique terms remained and were classified into four categories: *symptom description*, *pain location*, *consequences*, and *impact* (Fig. 2). One group identified comorbidities using the term “pain elsewhere/other joints,” but since this was deemed not specific enough to describe painful TMD per se, the term was excluded before categorization.

**Table 1:** Participants and contributors to the Global Burden of Orofacial Pain symposium on June 23rd, 2025

Chair	Corine Vissscher (Netherlands)	Justin Durham (UK)	Mark Drangsholt (USA)	Peter Svensson (Denmark/Singapore)	Anna Lövgren (Sweden)
Facilitator	Nikola Stanisic (Sweden)	Eunice Lua (Singapore)	Birgitta Häggman-Henrikson (Sweden)	Tessa Bijelic (Sweden)	Alicia Böthun (Sweden)
Participants	Malin Ernberg (Sweden) Daniele Manfredini (Italy) Roberto Rongo (Italy) Ambra Michelotti (Italy) Merel Verhoeff (Netherlands) Donald Nixdorf (USA) James Allison (UK)	Thomas List (Sweden) Evelina Nilsson (Sweden) Anna Colonna (Italy) Juan Fernando Oyarzo (Chile) Beth Groenke (USA) Chris Penlington (UK) Claudia Restrepo (Colombia)	Frank Lobbezoo (Netherlands) Giuliana Lunecke (Chile) Xin Xiong (China) Carolina Marpaung (Indonesia) Lene Baad-Hansen (Denmark) Mieszko Wieckiewicz (Poland) Oliver Schierz (Germany) Rosaria Bucci (Italy)	Laura Nykänen (Finland) Estephan Moana-Filho (USA) Maria Pigg (Sweden) Emma Beecroft (UK) Nikolaos Christidis (Sweden) Axel Kutschke (Sweden) Manolis Jusakos (Chile) Carolina Yanez (Germany)	Begoña Moreno (Chile) Nikolaos Nikitas Giannakopoulos (Greece) Chris Peck (Australia/Singapore) Andrzej Malysa (Poland) Draghici Raluca (Romania) Michalis Koutris (Greece/Netherlands) Daniela Gobi Goncalves (Brazil) Hedwig van der Meer (Netherlands)

**Table 2:** Existing lay descriptions that could be relevant for TMD pain

Sequela	Health status name	Parts of lay description that could work for OFP
Mild heart failure due to ischemic heart disease	Heart failure, mild	Easily tires with moderate physical activity
Uncomplicated diabetes mellitus type 1	Generic uncomplicated disease: worry and daily medication	Causes some worry, but minimal interference with daily activities
Other mild mental disorders	Anxiety disorder, mild	Feels mildly anxious and worried, which makes it difficult to concentrate, remember things, and sleep Tires easily
Pain due to caries of deciduous teeth	Dental caries, symptomatic	Causes some difficulty in eating
Severe neck pain	Neck pain, severe	Gets headaches Sleeps poorly and feels tired and worried Sleeps poorly
Difficulty eating due to edentulism and severe tooth loss	Severe tooth loss	Great difficulty in eating meat
Symptomatic medication overuse headache due to migraine	Headache, medication overuse	Has daily headaches, felt as dull pain and often lasting all day Takes medicine that provides little relief, but is needed to avoid worse symptoms
Symptomatic probable migraine	Headache migraine	Difficulty in daily activities
Symptomatic probable tension-type headache	Headache, tension-type	Difficulty in daily activities

The proposal to use “mild,” “moderate,” and “severe” to distinguish between different levels of severity was perceived to be appropriate, and all groups discussed these potential categories accordingly. In line with this suggestion, Table 3 shows how symptom severity can be reflected within these levels of painful TMDs. In general, pain duration, intensity, and frequency were suggested to be important dimensions to increase the granularity of the severity levels. None of the groups used AI tools to generate lay descriptions during the symposium because face-to-face discussions were prioritized during the allocated time slots. Thus, the group chairs used such tools after the main workshop. Table 3 presents the AI-derived lay descriptions. By merging the prioritized terms from the symposium with the AI-derived versions, the following lay description was suggested for TMD pain: “Pain in the jaw, face, cheeks, or around the ears, sometimes radiating to the temples or behind the eyes. The pain may be felt as dull, sharp, tense, or stiff, making chewing, talking, or opening the mouth difficult.” Proposed descriptions of the mild, moderate, and severe states are also presented in Table 3.

**Table 3:** Suggested terms of relevance and lay descriptions of painful TMDs, including specifications for mild, moderate, and severe states

	Suggested terms of relevance	Group-generated descriptions	AI-derived descriptions	Merged descriptions
Painful TMDs		Pain or discomfort in the jaw, face, or around the ears that can make it hard to chew, talk, or open the mouth. It may come and go or be constant	Pain in the jaw joint and the muscles used for chewing. It often causes jaw stiffness, difficulty opening the mouth, and pain when chewing or talking	Pain in the jaw, face, cheeks, or around the ears, sometimes radiating to the temples or behind the eyes. Can feel dull, sharp, tense, or stiff, making chewing, talking, or opening the mouth difficult
Mild	Uncomfortable, discomfort, pressure, mild version of “Difficulty eating, chewing, talking, chewing,” temporary/intermittent/occasional inconvenience/disability, comes and goes, stress Tension, pulling, tightness, stiffness, fatigue, tiredness, exhaustion, tiredness	Occasional jaw or facial discomfort that may feel dull or tiring, usually triggered by chewing or talking, but does not interfere much with daily activities	Occasional jaw pain. It is uncomfortable and makes chewing unpleasant, but does not prevent eating, speaking, or doing most daily activities	Occasional jaw or facial discomfort that may feel dull, tiring, or tight, with mild pressure, tension, or stiffness. Pain comes and goes, often triggered by chewing, talking, or stress, causing temporary inconvenience
Moderate	Dull, sore, difficulty eating, chewing, talking, chewing, inconvenience, distressing, frustrating Toothache, jaw cramping, jaw movement limitation, difficulty eating, limiting activity, can’t eat, talk, smile, can’t open mouth wide, bad sleep, fear of opening mouth, kinesiophobia	Recurring jaw or facial pain that can limit chewing or talking. It may feel constant or come and go, and it can cause frustration or worry during daily tasks	Frequent jaw pain that worsens with chewing or talking. It sometimes makes opening the mouth difficult, interferes with eating certain foods, and causes ongoing discomfort in daily activities	Recurring jaw or facial pain that may feel dull, sore, or cramping, sometimes like a toothache. It can limit chewing, talking, or opening the mouth, causing frustration, worry, or poor sleep, affecting daily activities
Severe	Throbbing, nutrition (can’t eat), disabling, distressing Productivity loss, scary, torture, stabbing, piercing, splitting, all-consuming, key focus, asocial activity	Persistent pain in the jaw or face that makes it hard to eat, speak, or sleep. It may cause emotional distress and greatly affects everyday life	Constant, strong jaw pain that spreads to the face, head, or neck. The pain makes eating, talking, and opening the mouth difficult, severely limiting daily activities and quality of life	Persistent, throbbing, or piercing pain in the jaw and face that makes eating, speaking, or sleeping very difficult. Pain is disabling, all-consuming, and distressing, severely limiting daily life

## Discussion

The main finding from this ongoing work on enabling the inclusion of TMD pain into GBD measures is that no existing lay descriptions directly capture the essence of experiencing painful TMDs. Such descriptions should reflect not only pain modified by function but also the consequences and impact of the pain. In addition to considering pain duration, used to distinguish acute from chronic pain and captured in GBD burden estimates through data on onset and remission, pain intensity and frequency appear to be key factors in categorizing mild, moderate, and severe states of painful TMD. Furthermore, the specific consequences and impact of painful TMDs should be incorporated into these levels of severity, as is the case in the clinical assessment of TMD impact with the Graded Chronic Pain Scale (GCPS) [26] and Jaw Functional Limitation Scale [27]. Here, we present the first step in providing a consensus-derived proposal for lay descriptions of painful TMD with grading into mild, moderate, and severe states.

Building on the rigorous work that has been carried out over the years to standardize the assessment and diagnosis of TMD [5, 22, 28], the field of OFP now has a solid foundation for the use of standardized, evidence-based criteria in the development of new terminology, including lay descriptions. In line with the current criteria, diagnostic outcomes remain binary in nature, yet the inclusion of both frequency and severity measures has been recommended [29].

In general, descriptions of pain duration, intensity, and frequency add important granularity to assessments of severity. Acute pain episodes, which are typically and by definition less than 3 months long, may reflect a burden with lower severity, whereas recurring pain suggests a moderate level of symptom burden. Chronic or persistent pain, on the other hand, is often related to more complex mechanisms and is therefore associated with higher severity, greater functional impact, and an increased need for clinical attention and a holistic approach in assessment [30]. The importance of adopting a biopsychosocial perspective in TMD pain assessment and management has been reflected in the dual-axis system, linking a clinical diagnosis with a psychosocial screening, and has been incorporated into TMD diagnostic criteria for more than three decades [5, 22, 28]. Despite long-standing availability, these criteria have largely failed to be adopted in data collection and clinical practice, and substantial inconsistencies persist across existing taxonomic frameworks. Alignment between ICD-11, ICOP-2 [31], and DC/TMD is therefore essential for future standardized assessment and meaningful data collection.

In the context of pain ratings in general, it has also been suggested that pain intensity may serve as an indicator of pain-related distress [32]. Applied to the context of TMD and OFP, this interpretation could provide an additional dimension to the current binary diagnostic framework, offering nuances in the understanding of symptom severity. In TMD and OFP, intensity levels below 4 on a numerical rating scale (NRS) are typically associated with mild distress, ratings between 4 and 6 with moderate distress, and scores of 7 or higher with severe distress [32]. By further building on pain intensity as a marker of severity, pain frequency may also serve as an indicator of the overall pain burden. In the GCPS [33, 34], pain intensity measured on an NRS scale reflects the magnitude of the pain experience, whereas pain frequency contributes to the assessment of pain-related disability and chronicity. Similarly, in the context of TMD screening, Fonseca’s questionnaire—one of the two available and validated screening systems for TMD in the general population [35]—captures both prevalence and severity, classifying symptoms as mild, moderate, or severe depending on their frequency [36]. According to the 3Q/TMD screening instrument, a pain frequency of once a week or more is associated not only with a painful TMD diagnosis [37] but also with higher pain intensity and a clear indication of a need for treatment [38]. These considerations of both pain frequency and pain intensity underscore how combining multiple dimensions of the pain experience can provide a more nuanced understanding of symptom severity, as reflected in our proposed lay descriptions for painful TMDs.

Accurate lay descriptions play a crucial role in estimating disability weights. When key health impacts that distinguish severity levels are omitted, the true burden of a condition can be seriously underestimated. For instance, in the most recent GBD disability weight estimation, the lay descriptions for hearing loss were revised to explicitly include social isolation as a health impact. This change alone increased the disability weight from 0.093 to 0.316 [19]. Therefore, our efforts for developing lay descriptions that fully capture the health impacts of painful TMDs across their severity spectrums are essential for generating reliable estimates of its true burden. In addition to painful TMDs, non-painful TMDs, including limitations in jaw opening as well as jaw catching and locking, may also contribute to disease burden. Consequently, from a policy perspective, developing lay descriptions that include all major OFP conditions including TMD, and with sufficient granularity provides an essential foundation for quantifying disability and informing efficient resource allocation. Doing so as a collaborative effort with OFP and TMD experts ensures clinical accuracy and relevance. Incorporating OFP more broadly into GBD metrics is critical due to the unique characteristics of different pain types, including variations in intensity, frequency, affected tissues, and impact on multiple and vital oral functions. This is important since standardized, comparable measures of OFP burden are crucial in recognizing its true impact and supporting the development of equitable healthcare policies and resource distribution worldwide.

Beyond their methodological role in GBD estimation, lay descriptions hold substantial value for patients, clinicians, and policymakers. From a patient perspective, clear and relatable wording can help transform OFP, including painful TMDs, from a condition often perceived as invisible—in particular, labeled by patients as an “invisible problem”—into something assessable and recognizable. Such a transformation is of special importance for chronic pain conditions, since early identification has been highlighted as a key factor in improving long-term prognosis [39]. Moreover, validation through identification and recognition has been described as one of the elements currently perceived to be lacking in the treatment of pain patients in healthcare, including dentistry [40]. Therefore, the construction of lay descriptions to be implemented in a broader perspective and in the real world may itself contribute to patient satisfaction and positively influence patient perception of care provision. A logical next step would be inviting patients with a broader spectrum of severity levels to review whether the proposed lay description accurately represents their lived experience.

From a clinical perspective, lay descriptions of painful TMDs can help healthcare professionals bridge the gap between technical diagnostic criteria and patients’ lived experiences. This is also in line with other initiatives within the field of painful TMDs, such as the recently published key points for clinical practice [41]. The translation from clinical assessments into clear, relatable language can capture not only the location but also the intensity, frequency, and functional impact of painful TMD. Since painful TMDs are often perceived by healthcare providers as challenging to manage [42, 43], and since this field lies at the interface between dentistry and medicine [44], the implementation of the work presented here can facilitate more effective communication and further support early recognition, validation, and shared decision-making. Therefore, future work should focus not only on interdisciplinary and transdisciplinary approaches, but also on involving other pain-related healthcare professionals, such as neuroscientists, neurologists, and anesthesiologists.

Although consensus was achieved in this work, some limitations should be acknowledged. Even though the symposium was announced and open to all IADR/ INfORM members, the workshop participants could not fully represent the diversity of perspectives across different backgrounds, such as all professions working with pain, world regions, and cultural contexts, which could reduce the generalizability of the proposed descriptions. Moreover, although the consensus process was structured and guided by IHME experts, it did not follow a formal Delphi methodology, which might have provided a stricter and more systematic approach to achieving consensus. Finally, the discussions and proposals of lay descriptions were conducted in English, which may have influenced the wording and interpretation, particularly given cultural and linguistic variations in how pain is expressed and understood. Therefore, translation and validation in other languages should be considered a next logical step.

## Conclusions

The lay description provides direction on how painful TMDs can be quantified in GBD estimates. Future studies, including those that incorporate patients’ perspectives, are essential to ensure alignment with lived experiences.

## Supplementary Information

Additional file 1. Invitation for the workshop held at the 2025 IADR general session.

Additional file 2: Table 1. Instructions for group discussions.

Additional file 3: Table 2. Crude list of terms derived from the discussions and classified into four categories.

## Data Availability

All data generated or analysed in this study are included in this published article and its supplementary information files.

## Abbreviations

DC/TMD: Diagnostic Criteria for Temporomandibular Disorders
GBD: Global burden of disease
GCPS: Graded Chronic Pain Scale
IADR: International Association for Dental, Oral and Craniofacial Research
ICOP: International Classification of Orofacial Pain
IHME: Institute for Health Metrics and Evaluation
INfORM: International Network for Orofacial Pain and Related Disorders Methodology
NRS: Numerical rating scale
OFP: Orofacial pain
TMD: Temporomandibular disorders
