# Supplementary material for: Lay descriptions of painful temporomandibular disorders—an international consensus proposal for Global Burden of Disease estimates
Source: BMC Med. 2026 Mar 17;24:165. doi: 10.1186/s12916-026-04790-3 (PMC12997997; doi:10.1186/s12916-026-04790-3)
Supplement: Supplementary file 1 — Additional file 1. Invitation for the workshop held at the 2025 IADR general session. [file 12916_2026_4790_MOESM1_ESM.pdf]

Satellite symposium proposal
IADR 2025

1. Proposal title: Joint action needed: Integrating orofacial pain into Global Burden measures

2. Proposal description:

The global burden of disease (GBD) initiative has been introduced as a systematic effort to estimate the magnitude of all major diseases, risk factors, and intermediate clinical outcomes in a highly standardized way and with the purpose to quantify health loss across regions over time. Despite being the third most common pain condition with a significant impact on the individual and the society, orofacial pain including Temporomandibular Disorder (TMD) pain is currently not included in global measures of health. Accurate measures of orofacial pain burden are therefore crucial to ensure that the impact of orofacial pain is recognized and addressed to achieve more equitable healthcare policies and resource allocation worldwide. This cutting-edge symposium on how we can join our actions to bring orofacial pain into global health discussions will be outlined and discussed at this satellite symposium.

3. Describe how this session will promote innovation: To enable estimations of the global burden of a condition, a disability weight together with a lay description of a condition are key requirements. By providing such lay descriptions on behalf of INfORM, we can significantly contribute to orofacial pain and TMD being recognized and prioritized in global health discussions.

4. Sponsoring scientific group(s)/network(s):

International Network for Orofacial Pain and Related Disorders Methodology (INfORM) (Sponsor);

Neuroscience (Co-Sponsor);

Oral health research group (Co-sponsor).

Educator/Clinician track: Clinician track

5. Learning objectives:

Knowledge and understanding of:

- The overall concepts of GBD studies including global burden of pain
- The current level of knowledge and need for next steps to incorporate TMD and orofacial pain into GBD measures.

- The importance of establishing disability weights and connected lay descriptions

6. People:

Organizers: Peter Svensson (National University of Singapore, Singapore), Mark Drangsholt (University of Washington, USA) and Anna Lövgren (Umeå University, Sweden).

Chairpersons: Justin Durham, Corine Visscher, Peter Svensson, Mark Drangsholt, Anna Lövgren

Speaker 1: Peter Svensson

15 minutes – Global Burden of Pain

Speaker 2: Mark Drangsholt

15 minutes – The prerequisites for GBD estimates for orofacial pain including data collection on incidence, prevalence, and risk factors.

Speaker 3: Anna Lövgren (Umeå University, Sweden)

15 minutes – The importance of a disability weight and available methods

Speaker 4: Suzie Bergman, stakeholder perspective from being a patient

15 minutes – Why is healthcare disjoint – the patient perspective of incorporating orofacial pain into healthcare.

General Discussion: moderated by chairpersons, with organizers, all speakers, and attendants

60 minutes – What lay descriptions should be connected to orofacial pain and TMD respectively?

30 minutes – Summary and future directions including establishing a taskforce

Keywords: Health services research, Global Burden of Disease, orofacial pain, temporomandibular disorders

7. Miscellaneous: The afternoon on the day before the official meeting i.e., Tuesday 24th of June 2025.

8. Audio recording: Agreement obtained from all speakers.

9. Room set-up requests: Data projector
