# Supplementary material for: Lay descriptions of painful temporomandibular disorders—an international consensus proposal for Global Burden of Disease estimates
Source: BMC Med. 2026 Mar 17;24:165. doi: 10.1186/s12916-026-04790-3 (PMC12997997; doi:10.1186/s12916-026-04790-3)
Supplement: Supplementary file 2 — Additional file 2: Table 1. Instructions for group discussions. [file 12916_2026_4790_MOESM2_ESM.pdf]

Instructions for group discussions
Lay descriptions, IADR 2025

Activity	
Step 1<br><br>Brainstorming<br>Key Words	Work in **pairs** to identify words that describe **TMD pain**.<br><br>Write each word on a **separate post-it note**.<br><br>Share in the group.
Step 2<br><br>Review Existing<br>Lay Descriptions	Review the **existing lay descriptions** available (e.g., for neck pain, back pain, toothache).<br><br>Discuss if any could apply to **TMD pain**.
Step 3<br><br>Prioritize and Categorize Words	As a group, **select and prioritize** the most relevant words from the brainstorm.<br><br>Organize words into a **hierarchical list**.<br><br>Introduce the health states: **mild, moderate, severe**.<br><br>Discuss whether certain words relate more strongly to a specific severity level.
Step 4<br><br>Create Lay Descriptions	Use the prioritized words to draft **lay descriptions** of TMD pain.<br><br>In the next step. Differentiate into:<br><br>- Mild<br>- Moderate<br>- Severe
Step 5<br><br>Use AI tools	Use AI tools to develop lay descriptions for TMD pain and for differentiating into mild, moderate and severe.<br><br>Compare and discuss similarities and differences between the versions.<br><br>Decide on the most relevant ones.
