# Supplementary material for: Lay descriptions of painful temporomandibular disorders—an international consensus proposal for Global Burden of Disease estimates
Source: BMC Med. 2026 Mar 17;24:165. doi: 10.1186/s12916-026-04790-3 (PMC12997997; doi:10.1186/s12916-026-04790-3)
Supplement: Supplementary file 3 — Additional file 3: Table 2. Crude list of terms derived from the discussions and classified into four categories. [file 12916_2026_4790_MOESM3_ESM.docx]

Additional file. . Crude list of terms derived from the discussions and classified into five categories. Terms prioritized are shown in bold.

|  | Words/ terms |
| --- | --- |
| Total number of words derived from the brainstorm | Pressure, tired, fatigue, dysesthesia, sore, tired, mild or sharp,  Jaw pain, provoked, fear, anxiety, face, chronic, continuous, muscle and joint, emotional distress, difficulty chewing, spread, distressing, stress, frustrating, anxiety, negative mood effect, sadness, distressing, anxiety, depression, joint noises, crackling, rustling, intense, pain, jaw joint noises, intermittent, constant, exhausting, pop, click, crack, noise, unrelenting, noises – click, grating, catching, clicking, sounds, chewing – difficulties, talking, eating, chewing, nutrition (can’t eat), difficulty eating, drinking, talking, chewing, problems while eating, cannot open my mouth, can’t open mouth, dysfunction, trismus – limitation of mouth opening, limitations of jaw opening, wasted jaw function, face/facial, mouth, teeth, head, jaw, pressure → tooth, waking up with pain, pain elsewhere other joints, unpredictable, throbbing, ache, aching, tiredness, stiffness, sore, dull, uncomfortable, discomfort, disruptive, difficulty doing usual activities – work, social, family, disabling, disable, relationship difficulties, inconveniences (missed days works, school for appointments, feeling unwell…), hard to think/focus, disabling, limits social opportunities, disability → expensive → disability, memory problems, handicap, limitation in daily functioning, disable, twisting, nagging, dull, dull and achy, sour, sharp, maddening, bothersome, pain when waking up, painful clicking, jaws, temples, behind eyes, face, cheeks, temples, in front of ears, ears, tooth pain, headache, sharp pain in front of ear, pain in cheek area, radiating pain from cheek, uncomfortable, feeling stiff, feeling tense in jaws, jaw fatigue, ear ringing, tinnitus, fatiguing, fatigue with jaw function, exhausting, difficulty chewing,  Difficulty talking, difficulty speaking, difficulty eating, difficulty jaw opening, difficulty yawning, interferes with jaw use, interferes with intimacy/kissing, limited jaw opening, cannot easily chew, chewing, impairment, disturbed sleep, difficulty sleeping, difficulty concentrating, cannot think, cannot be with people, cannot be social, socially limiting, stress, bite is off, muscle, temple, cheeks, jaws, jaw joint, masticatory muscle, in front of the ear, jaw pain,  Pain in the face, ear, tension, tight, tender, sore, pressing, stabbing,  Aching, hot/deep, throbbing, fatigue, pulsing, burning, heaviness, tiresome,present all the time, draining, tiring, fatigue, frustrating, exhausting,flare ups, cyclic, acute, chronic, all aspects of daily life, pain doesn’t allow me to, limiting,difficulties using the jaw, difficulties concentrating, sleep disturbance/difficulties, ache, annoying, cramping, dull, fatigue, fear to open, jaw cramping, jaw movement pain, jaw muscle fatigue, jaw pain, muscle fatigue, numbness, piercing, pulling, recurring, scary, sore, splitting, stiffness, suan (sour in singapore), temple pain, tension, tightness, tiredness, toothache, torture, affection limitations, afraid of activities, avoid family, avoid social/family meetings, bad sleep, can’t talk for long time, cannot kiss, cannot open mouth wide, can’t eat, can’t smile, can’t talk, change in eating pattern, communication, cutting, difficulty eating food, eating pattern, exhausting, focus, jaw movement limitation, key focus, kinesiophobia, laughing, limitation with communication, limited social interaction, limiting aspects of life, movement limitation, productivity loss, stabbing |
| Words deemed not relevant | noises – click, grating, catching, clicking, sounds, joint noises, jaw joint noises, pop, click, crack, noise, ear ringing, tinnitus  Pain elsewhere (other joints) |
| Remaining words after removal of duplicates and per categories | Pain location  **Behind eyes**  **Cheeks**  **Ears, In front of ears**  **Face**  **Head**  **Jaw**  **Mouth**  Muscle and joint  **Radiating pain from cheek**  **Temples**  **Teeth**  Symptom description  **Ache**, Aching  Acute  Annoying  **Bothersome**  Burning  Crackling  **Chronic**, Continuous, **Constant**, **Persistent**, Present all the time  Cramping  **Cutting**  Cyclic, Flare ups, **Fluctuating**, **On and off**, Recurring  Deep  Disruptive  Disable  Discomfort, **Uncomfortable**  **Dull**  Draining  Dysesthesia  Exhausting, **Exhaustion**  **Fatigue**, Fatigue with jaw function, Tired  Fear to open  Frustrating  **Headache**  Heaviness  Hot  Intense  Intermittent  **Maddening**  Mild  **Nagging**  Numbness  Piercing  Pressing, Pressure  **Pulling**  Pulsing  Provoked  Rustling  Scary  **Sharp**  Splitting  Spread  Stabbing  **Stiffness,** **Feeling stiff**  Tender  **Tension**, **Feeling tense**  Throbbing  **Tightness**  **Tiredness,** Tiresome, **Tiring**  Torture  **Twisting**  Unpredictable  Unrelenting  Stiffness  **Sore, Sour**  Consequences  Difficulty **chewing**, **eating,** drinking, **talking/speaking**, smiling, laughing, Affection limitations  Change in eating pattern  Cannot open my mouth, Dysfunction, Trismus – **Limitation of mouth opening/jaw opening**  Wasted jaw function  **Difficulty jaw opening**, **yawning**  Interferes with jaw use  Interferes with intimacy/kissing  **Pain when waking up**  Bite is off  Kinesiophobia  Impact  All aspects of daily life  Productivity loss  **Anxiety**  Negative mood effect  **Fear**  Emotional distress, Distressing, Stress  **Frustrating**  Hard to think/focus  Disabling  **Limiting**  Limits social opportunities, Limitation with communication, Limited social interaction, Limiting aspects of life  Disability → expensive → disability  Memory problems  Sadness  Depression  **Difficulty doing usual activities** – work, social, family,  **Disabling**, Disable  Relationship difficulties  Inconveniences (missed days works, school for appointments, feeling unwell…)  Handicap  Limitation in daily functioning  Impairment  Difficulty sleeping, **Disturbed sleep**  **Difficulty concentrating**, **Cannot think**  **Upsetting** |
